# Older Americans Month — May 2014

**Published:** 2014-05-02

Each May, the nation celebrates Older Americans Month to recognize older persons for their contributions and provide information to help them stay healthy and active. This year’s focus is injury prevention, with the theme “Safe Today. Healthy Tomorrow.”

Injuries and violence are serious threats to the health of persons aged ≥65 years. Unintentional injuries among this population result in approximately 48,550 deaths annually (1). Falls are the leading cause of fatal and nonfatal injuries among older adults (2). About a third of those aged ≥65 years fall each year, resulting in costs of nearly $30 billion annually (1). Older adults are also at higher risk for traumatic brain injury and injuries associated with residential fires, abuse and maltreatment, and suicide (3).

To improve older adult health, CDC works to reduce risk factors for injuries and ensure widespread adoption of effective injury prevention strategies. Information on fire safety and interventions to prevent falls among older adults, for example, can be found at http://www.cdc.gov/homeandrecreationalsafety. Information and resources (including posters and sample articles) about Older Americans Month are available at http://www.acl.gov/newsroom/observances/oam/index.aspx.
